# A thrombomodulin-like gene is crucial to the collective migration of epibolic blastomeres during germ layer formation and organogenesis in zebrafish

**DOI:** 10.1186/s12929-019-0549-2

**Published:** 2019-08-26

**Authors:** Gang-Hui Lee, Chia-Lin Chang, Wen-Tai Chiu, Tsun-Hsien Hsiao, Po-Yuan Chen, Kuan-Chieh Wang, Cheng-Hsiang Kuo, Bing-Hung Chen, Guey-Yueh Shi, Hua-Lin Wu, Tzu-Fun Fu

**Affiliations:** 1 The Institute of Basic Medical Science College of Medicine, National Cheng Kung University, Tainan, Taiwan; 2 International Center for Wound Repair and Regeneration, National Cheng Kung University, Tainan, Taiwan; 3 Department of Biochemistry and Molecular Biology, National Cheng Kung University College of Medicine, Tainan, Taiwan; 4 Cardiovascular Research Center College of Medicine, National Cheng Kung University, Tainan, Taiwan; 5 Department of Biomedical Engineering College of Engineering, National Cheng Kung University, Tainan, Taiwan; 6 Department of Medical Laboratory Science and Biotechnology, National Cheng Kung University College of Medicine, Tainan, Taiwan; 7 Department of Food Safety/ Hygiene and Risk Management College of Medicine, National Cheng Kung University, Tainan, Taiwan; 8 Department of Pharmacy College of Pharmacy and Science, Chia Nan University of Pharmacy and Science, Tainan, Taiwan; 9 Department of Biotechnology, Kaohsiung Medical University, Kaohsiung, Taiwan; 10 The Institute of Biomedical Sciences, National Sun Yat-sen University, Kaohsiung, Taiwan

**Keywords:** Thrombomodulin, Collective cell migration, Cytoskeleton, Germ layers formation, Organogenesis, Zebrafish

## Abstract

**Background:**

Thrombomodulin (TM), an integral membrane protein, has long been known for its anticoagulant activity. Recent studies showed that TM displays multifaceted activities, including the involvement in cell adhesion and collective cell migration in vitro. However, whether TM contributes similarly to these biological processes in vivo remains elusive.

**Methods:**

We adapted zebrafish, a prominent animal model for studying molecular/cellular activity, embryonic development, diseases mechanism and drug discovery, to examine how TM functions in modulating cell migration during germ layer formation, a normal and crucial physiological process involving massive cell movement in the very early stages of life. In addition, an in vivo assay was developed to examine the anti-hemostatic activity of TM in zebrafish larva.

**Results:**

We found that zebrafish TM-b, a zebrafish TM-like protein, was expressed mainly in vasculatures and displayed anti-hemostatic activity. Knocking-down TM-b led to malformation of multiple organs, including vessels, heart, blood cells and neural tissues. Delayed epiboly and incoherent movement of yolk syncytial layer were also observed in early TM-b morphants. Whole mount immunostaining revealed the co-localization of TM-b with both actin and microtubules in epibolic blastomeres. Single-cell tracking revealed impeded migration of blastomeres during epiboly in TM-b-deficient embryos.

**Conclusion:**

Our results showed that TM-b is crucial to the collective migration of blastomeres during germ layer formation. The structural and functional compatibility and conservation between zebrafish TM-b and mammalian TM support the properness of using zebrafish as an in vivo platform for studying the biological significance and medical use of TM.

**Electronic supplementary material:**

The online version of this article (10.1186/s12929-019-0549-2) contains supplementary material, which is available to authorized users.

## Background

Thrombomodulin (TM), a type I transmembrane glycoprotein expressed on the surface of endothelial cells, has long been known for its anticoagulant activity. TM binds with thrombin, the key protease activating fibrinogen to fibrin for clot formation, and accelerates the activation of protein C. The activated protein C (APC) inactivates coagulation factors Va and VIIIa, and thereby stops the propagation of coagulation cascade [1, 2]. TM–thrombin complex also activates thrombin-activatable fibrinolysis inhibitor, endowing TM with anti-fibrinolytic activity [3]. The membrane-bound TM can be cleaved enzymatically and released into circulation as soluble fragments. Heterogeneous soluble TM is found normally in plasma, urine and synovial fluid, but also correlated to the activity of diseases [4, 5]. Elevated plasma levels of TM had been associated with cardiovascular disease, diabetes, ischemic and/or inflammatory endothelial injuries [6–8]. Studies suggested that TM might serve as a potential index for the progression and metastasis of cancers in patients with lung cancers, gastric cancers and pancreatic cancers [9]. The beneficial effects provided by recombinant TM in relieving abdominal aortic aneurysm further support the pathological significance and therapeutic potential of TM, highlighting the importance of TM in both healthy and diseases states. In addition to its role in hemostasis, TM displays multifaceted activities and exerts profound impacts on several biologic processes. TM had been found to express in various types of cells, including astrocytes, keratinocytes, osteoblasts, neutrophils, chondrocytes, alveolar epithelial cells and ciliary non-pigmented epithelial cells of the eyes, supporting for the potential activities of TM other than thrombin-APC dependent anticoagulant [[10] and references therein]. The embryos of homozygous TM-knockout mice were less-developed and died before a functional cardiovascular system was established [11–13]. Reestablishment of TM expression in extra-embryonic tissues rescued TM-null embryos from early lethality, supporting a role for TM in the development of non-endothelial tissues [14]. TM is also found to be involved in inflammation [15–19], angiogenesis [20–22], cell adhesion [23] and wound healing [24–26]. The activity of TM in modulating cell adhesion empowers this molecule an important player in collective cell migration, the phenomenon of massive cell movement during embryogenesis and cancer metastasis [27, 28]. These activities, especially in modulating cell migration, endow TM a vital role in the very early embryogenesis where many important physiological pathways are activated and operating concurrently to allow the massive movement of primordial germ cells to occur and the germ layers to be formed. However, how TM functions and contributes to these biological processes in both physiological and pathological states remain incompletely understood to date. Therefore, further investigation on TM becomes essential not only for improving our knowledge about TM but also for the best use of this molecule in medical practice, as well as the medication-safety concerns.

Studies with model animal provide inimitable information on the fundamental mechanisms of biological processes and diseases, which could not be comprehensively revealed with cultured cells. This is best exemplified by the studies on collective cell behaviors and cell-microenvironment interactions, two pivotal processes during embryogenesis. Currently, most of the in vivo studies related to TM were conducted mainly on rodents, with which much valuable information has been revealed. Nevertheless, deciphering the role of TM in early embryonic development in mammals could be challenging due to the limitation of intrauterine development and the early embryonic lethality occurring to the TM-deficient transgenic animals [12]. With the combined advantages of in vivo complexity and in vitro convenience of high-throughput screening, zebrafish is a vertebrate model prominent for research on molecular function, diseases mechanism, developmental biology and drug discovery. Zebrafish embryos are transparent and develop externally following fertilization, allowing for a real-time and continuous observation starting from the first cell division for the cellular behavior during embryogenesis. The external fertilization/development of zebrafish embryos also make them easily accessible to molecular manipulation for examining the activities of molecule/pathway with gain- and lost-of-function strategies. As in mammal, zebrafish possess a closed cardiovascular system with conserved cardiac components including atria, ventricles, cardiac valves and cardiac conduction system [29]. The genetic programs and molecular mechanisms involving in most biological processes in zebrafish are highly conserved [29–31]. More importantly, zebrafish embryos/larvae can survive till 5 to 7 day-post-fertilization (dpf) without a functional circulation system, allowing for the investigation and intervention that might lead to congenital circulation defects. Zebrafish is especially promising in repurposing of FDA-approved medications [32]. In addition, FDA itself has begun to invest heavily in zebrafish research “(https://www.fda.gov/consumers/consumer-updates/zebrafish-make-splash-fda-research; https://www.nature.com/articles/laban.1236)”. Currently, zebrafish TM remains an unexplored territory.

To further understand the significance of TM at the earlier stages of life, we explore the role of TM in the primordial germ cells movement during epiboly in developing zebrafish embryos. In this study, we report a zebrafish TM-like molecule displaying structural and functional comparability to human ortholog. The anti-coagulant activity and the expression of this TM-like molecule in zebrafish embryos were examined. The evidence to show that this TM-like protein co-localizes with cytoskeleton and modulates collective cell migration in vivo during embryogenesis are also provided and discussed.

## Materials and methods

### Materials

Polymerase chain reaction (PCR) primers were from MDBio, Inc. (Taipei, Taiwan). Restriction enzymes were purchased from New England BioLabs, NEB (Hitchin, UK). Texas-Red dextran was purchased from Thermo Fisher (Waltham, USA). In vitro transcription kit, anti-DIG antibody, and NBT-BCIP used for WISH were purchased from Roche (Basel, Switzerland). Horse radish peroxidase (HRP)-conjugated goat anti-mouse IgG and anti-human TM antibodies were from Santa Cruz Biotechnology (Santa Cruz, USA). All other chemicals, including buffers, N-phenylthiourea and o’-dianisidine were purchased from Merck (Kenilworth, USA).

### Fish line and maintenance

The AB strain zebrafish, the transgenic line Tg (*Fli1*:eGFP), which express green fluorescent protein in vessels, and Tg (*mpx*:eGFP), which express green fluorescent protein in neutrophils, were purchased from Taiwan Zebrafish Core Facility [33–35]. Zebrafish were maintained in a 14–10 h light-dark diurnal cycle at 28 °C following the standard husbandry procedures [36]. Embryos were staged according to Kimmel et al. [37]. The animal studies and all procedures for handling zebrafish and embryos, including breeding and maintenance of fish and sample collection, were approved by Affidavit of Approval of Animal Use Protocol of National Cheng Kung University (IACUC Approval NO. 103218).

### Cloning for zebrafish TM-like cDNA

The primer pairs designed based on the nucleotide sequences available in GenBank (GenBank No.MF 694564 and MF 694565, respectively) were used to PCR amplify the complete sequence of TMs from zebrafish cDNA library. The PCR amplified products were TA-cloned into pGEMTeasy plasmid (Promega) and sub-cloned into peGFP-N1 between XhoI and SacII sites. The primer and 5′-AAACCTTTGATCGGTGTTGG-3′(reverse) for zebrafish TM-a; 5′-TCTTTAGGCAGAGAGACCACAA-3′(forward) and 5′-CAAATTGGGGCATACTGGTC-3′ (reverse) for zebrafish TM-b. For constructing the clones expressing zebrafish TM-a or zebrafish TM-b fused with eGFP, PCR with 2 different sets of primers were performed using the above-mentioned clones as templates. The primer sequences are: 5′-CCGCTCGAGATGAGAGAGCTCGTAATGGCGCTGGCG-3′ (forward) and 5′-TCCCCGCGGAATGTCTCTCTTTAAATCCCTGTTGGTACTTGG-3′ (reverse) for TM-a; 5′-CCGCTCGAGATGGGACAAAGCTTGCAGGAGC-3′ (forward) and 5′-TCCCCGCGGAGCCTGAGATTTTGCTGTGCTTGATTTTTC-3′ (reverse) for TM-b. The two restriction enzyme sites for XhoI or SacII (underlined) were introduced for the convenience of subsequent sub-cloning. Successful cloning was confirmed by restriction enzyme digestion and DNA sequencing.

### Zebrafish TM expression

Total RNA of embryos at indicated stages were performed with TRIzol reagent (Life technologies). cDNA was synthesized by reversed transcription reaction with MMLV-reversed transcriptase system (Promega). RT-PCR and quantitative real-time PCR (qPCR) were performed following the manufacturer’s instructions (Roche Inc., Basel, Swiss) using the same primer pairs:5′-TCTGTCAGCCGGGATTTAAG-3′ (forward) and 5′-GTGTTCACACGGTCCTGTTG-3′ (reverse) for zebrafish TM-a;5′- AATGCATGGAAGGAAAGTGC-3′ (forward) and 5′-TGCTTGGTGCATCTGTTAGC-3′ (reverse) for zebrafish TM-b (Applied Biosystems 7500, Thermo Fisher). These primers were designed based on the nucleotide sequences available in GenBank (GenBank No. MF 694564 and MF 694565, respectively). The primer 5′-AGACATCAAGGAGAAGCTGTG − 3′(forward) and 5′-TCCAGACGGAGTATTTAC − 3′(reverse) are for zebrafish β-actin (internal control). All samples were analyzed with a Real-Time PCR Detection System (Roche Molecular Systems, Inc) and SYBR FAST qPCR Master Mix (KAPA Biosystems). The data obtained were normalized with β-actin at the indicated stages and zTM-a at 0.17 dpf, sequentially, by using ΔΔCt method. Western blot analysis was performed as previously described using the antibodies against α-tubulin (sigma# T5168), GFP (GTX21218) and human TM (SC-13164).

### TM-b knockdown, expression and rescue

The sequences of zebrafish TM-MOs designed by the manufacturer (Gene-Tools, LLC, Philomath, OR), based on the zebrafish TM-a and TM-b mRNA sequences we provided, were: 5′-TGACCAGCGAGCATTTCACGGGTCT-3′ for TM-a, 5′- AGAAAAGACAGGGATACACTGCACA-3′ for TM-b and 5′-AGAAtAcAgAGGcATACACTcCACA-3′ for zebrafish TM-b mismatch MO. Scrambled MO mixture containing 4^25^ different nucleotide sequences was used as a standard control MO. For most microinjection, approximately 4.6 nl of solution containing zebrafish TM-MO and/or plasmids expressing thrombomodulin were injected into embryos at 1 to 2-cell stages to reach the desired concentrations (for knockdown: zebrafish TM-a MO, 0.92 pmol/embryo; zebrafish TM-b MO, 0.46 pmol /embryo; zebrafish TM-b mismatch MO, 0.92 pmole/embryos; for rescue or expression: pcDNA3.1-hTM, 100 pg/embryo; hTM truncated construct pEGFP/N1-hTM△C, 200 pg/embryo; pEGFP/N1-zTM-a, 200 pg/embryo; pEGFP/N1-zTM-b, 200 pg/embryo), unless indicated otherwise. Embryos injected with Danieu’s buffer and standard control MO served as injection controls. All reagents for microinjection were dissolved in degassed and RNase free Danieu’s buffer to make proper stock solutions.

### Hemostasis

Zebrafish larvae at 3dpf (days post fertilization) were injected with Texas-Red dextran, with/without the tested reagents, at common-cardinal vein. After 30-mins incubation, larval tail was amputated and the blood leakage from the wound was recorded 5 min after tail removal. For those larvae with altered TM expression, embryos were injected with TM-MOs and/or plasmids containing human TM coding sequences at 1 to 2-cell stages and grown to 3 dpf for the test.

### Thrombomodulin in vitro activity assay

The activity assay was performed following the protocol described by Huang [38]. Briefly, HEK 293 T cells were transfected with plasmids encoding the tested proteins including peGFPN1 (for eGFP), peGFPN1-hTM (for hTM-eGFP fusion protein), peGFPN1-zTM-a (for zTM-a-eGFP fusion protein) and peGFPN1-zTM-b (for zTM-b-eGFP fusion protein) with lipofectamine 3000 (Invitrogen). After washing thoroughly with PBS and Tris buffer sequentially, cells were incubated in reaction buffer containing antithrombin at 37 °C for 30 min. The activity of activated protein C was measured by adding the substrate peptide-nitroanilide diacetate at 37 °C and incubated for another 30 min before monitoring the absorbance at 405 nm on an ELISA reader.

### Whole-mount in situ hybridization and histochemical staining

The whole-mount in situ hybridization (WISH) with digoxigenin (DIG)-labeled riboprobes was performed following the protocol described by Jowett (2001) and Thisse (1993) [39, 40]. The samples for WISH were prepared following the protocol described previously [41]. Riboprobes were generated by in vitro transcription in the presence of digoxigenin-11-UTP from linearized plasmid template. The probe used for *TM-b* was generated from a linearized plasmid containing the partial coding sequences and 3′ UTR of zebrafish TM-b. Cryosectioning was performed following the protocols in the Zebrafish Book and as previously described [36, 42].

### Blood flow and cardiac function

Larva at 3 dpf was mounted in 3% methylcellulose and video-recorded for the blood flow in caudal vein (60frames/second) under a transmitted-light stereomicroscope (Leica, MDG28) equipped with a digital single-lens reflex camera (Canon, EOS 550D). The velocity of blood flow was estimated from the traveling distance of a same red blood cell captured in a serial video stills with the software Celltracker [43] on MATLAB R2015a system. The cardiac stroke area, ejection fraction and cardiac output of larvae were calculated with the following equations: Stroke area = end diastolic area − end systolic area; Ejection fraction = stroke area / end diastolic area; Cardiac output = stroke area × heart rate. End-systolic area and end-diastolic area of heart were measured from the bright field time lapse image sequence analysis on a serial video stills with the software ImageJ, an open platform for scientific image analysis (https://imagej.net/ImageJ).

### Hemoglobin staining

Hemoglobin was stained with o’-dianisidine solution as previously described [44]. In brief, larvae at 3 dpf were anesthetized with 0.016% tricaine and incubated in 0.6 mg/ml o’-dianisidine with 0.01 M NaOAc, 0.65% H_2_O_2_ and 40% EtOH for 15 min. Larvae were washed twice with 1× phosphate-buffered saline (PBS), fixed in 4% paraformaldehyde (PFA) and observed under a light microscope.

### Single blastomere migration

The plasmid encoding a green fluorescence protein (pEGFP-N1) was injected into one single cell of either control embryos or TM-b morphants at 64-cells stage. The blastomeres showing comparable fluorescence intensity were selected and continuously recorded from 6 hpf to 7 hpf under a fluorescence dissecting microscope (Leica). The migration of the fluorescent blastomeres was characterized by analyzing the serial images with the software CellTracker [43] on MATLAB R2015a system.

### Yolk syncytial layer (YSL) staining

YSL vital staining was performed as previously described with minor modifications [45]. Briefly, zebrafish embryo at 4hpf was injected with 2.3 nL of 0.25 mM SYTOX-Green at the junction of blastoderm and yolk, incubated at 28.5 °C for 2 h and observed under a fluorescence dissecting microscope.

### Larval spontaneous movement

Embryos at 24 hpf were video-recorded under a dissecting light microscope for 5 min. All episodes of movement for each embryo were counted and analyzed with the tracking software EthoVision XT (Version 12, Noldus).

### Lateral line staining

Lateral lines were visualized by staining the hair cells in lateral line neuromasts with 4-(4-diethylaminostyryl)-N-methyl pyridinium iodide (4-Di-2-ASP) for 10 min and observed under a fluorescence dissecting microscope (excitation 488 nm, emission 607 nm).

### Whole mount cytoskeleton staining

The whole mount cytoskeleton immunofluorescent staining was performed referencing the protocol described previously [41]. Fixed embryos were washed in PBS and dechorionated before incubated in PBDTx for 1 h and followed by a 4-h incubation in blocking buffer (PBS, 1% BSA, 10% goat serum, 1% DMSO, 0.1% Triton X 100). Embryos were incubated in Alexa Flour 594 Phalloidin (Invitrogen) for F-actin or primary antibody for α-tubulin overnight at 4 °C and deyolked on slides before mounted with cover slips. The immunofluorescent embryos were observed under a laser scanning confocal microscope (Olympus, FV1000). The co-localization of TM-b with cytoskeleton was analyzed by the FV10-ASW software (version 4.2a, Olympus).

### Statistical analysis

The probability value (*P* value) was calculated with Mann-Whitney nonparametric U test for morphological comparison and Student’s t test for the rest at 95% confidence intervals using software GraphPad Prism 5 (GraphPad Software; San Diego, CA). A P value less than 0.05 was considered statistically significant.

## Results

### The zebrafish TM-like proteins display structural conservation during evolution

Among the several cDNAs obtained by blast-searching with human thrombomodulin (hTM) peptide sequence in the on-line protein data bases, two cDNAs (ENSDARG00000092470 and ENSDARG00000077938) with the highest rankings of homology were cloned and studied. Gene structures and sequence analyses on the two cloned cDNAs showed that the full-length zebrafish TM-like protein-a (TM-a) is transcribed from a gene with two exons located on chromosome 20 and encodes a polypeptide of 517 amino acids in length. The full-length zebrafish TM-like protein-b (TM-b) is transcribed from a gene with single exon, as observed for human TM, located on chromosome 21 and encodes a polypeptide of 668 amino acids in length. It should be noted that a thrombomodulin (*thbd*) gene, located on chromosome 20, is annotated in ZFIN (http://zfin.org/ZDB-GENE-110411-281). Comparing the primary sequences of these three protein molecules revealed a 96 and 20% identity for TM-a and TM-b, respectively, with Thbd (Additional file 1: Figure S1). It is likely that TM-a and *thbd* were variants of the same gene. On the other hand, it is possible that TM-b and *thbd* are different genes, since they are located on different chromosomes, as well as the prominent dissimilarity in their primary structures.)

**Fig. 1:**
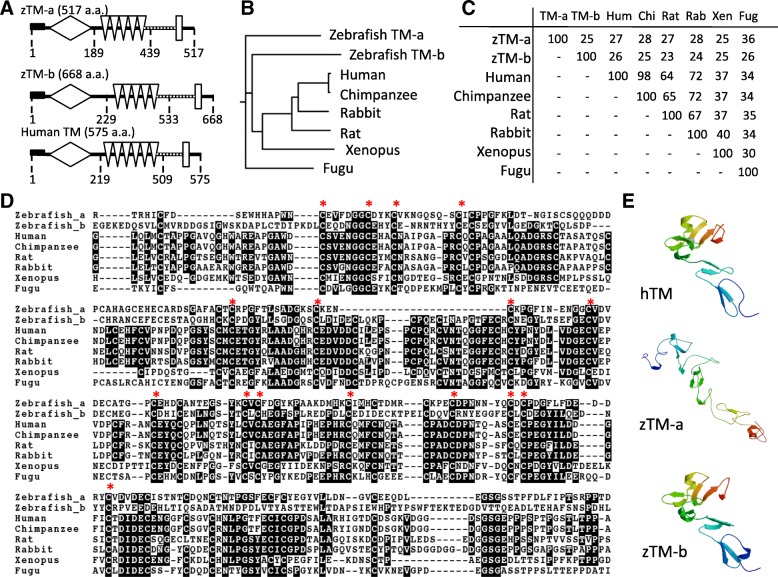
Structural and phylogenic comparison of zebrafish TM-a and TM-b. The amino acid sequences of TMs from the indicated species were analyzed and compared for functional domains and evolutionary trace. **a** The domain structures of zebrafish TM-a and TM-b were predicted with on-line software (SMART: http://smart.embl-heidelberg.de/) and compared with human TM. The depicted functional domains include: signal peptide (black oblong), c-type lectin domain (white rhomboid), EGF-like domain (white inverted triangles), an O-glycosylation site-rich region (dotted-line) and transmembrane domain (white oblong). **b** Clustering algorithm shows the evolutionary relationships of TMs among the compared species (ClustalW, SDSC Biology WorkBench http://workbench.sdsc.edu. **c** The similarity and percent of identity among the TM primary structures of the selected mammalian and aquatic species were compared. **d** The peptide sequences of TM EGF-like domains from the indicated species were aligned. The shaded letters indicate the amino acids that are identical among compared species. The residues indicated by asterisks are the cysteine residues participating in disulfide bonds and conserved during evolution. **e** The 3D structures of zTM-a (aa299 – aa410) and zTM-b (aa339 – aa452) were modeled with the on-line software SWISS-MODEL using the partial 3D structure of human TM (aa364 – aa480) available in the database as template. The compared peptide sequences include: Zebrafish TM-a (MF 694564); Zebrafish TM-b (MF 694565); Human TM (NP_000352); Chimpanzee TM (XP_003829427); Rat TM (NP_113959); Rabbit TM (NP_001075613); Xenopus TM (XP_002936969); Fugu TM (XP_003971425)

Both domain structure prediction and phylogenetic analysis revealed structural similarity and evolutionary clustering between the two zebrafish TM-like proteins and other TM orthologs (Fig. 1a and b). Human TM consists of 575 amino acids with 5 domains: Domain 1 (D1), an NH_2_-terminal lectin-like region; D2, an epidermal growth factor (EGF)-like domain; D3, an O′-glycosylation site-rich region; D4, a transmembrane domain; and D5, a cytoplasmic tail domain [10]. The characteristic structural domains identified in human TM, including the c-type lectin head at the N-terminus, the transmembrane region at the C-terminus, five epidermal growth factor-like (EGF-like) domains and a serine/threonine-rich spacer, were also predicted for both zebrafish TM-a and TM-b (Fig. 1a). The peptide sequences alignment for the EGF-like domains showed that zebrafish TM-a and TM-b are 27 and 26%, respectively, identical to that of human TM (Fig. 1c). Approximately 44% of the conserved cysteine residues involved in the formation of disulfide bonds crucial to the tertiary structure of mammalian TM are identified in the EGF-like domains of both zebrafish TM-a and TM-b (Fig. 1d). In addition, the motif crucial for TM interacting with ezrin/actin identified in human TM (R_540_K_541_K_542_) was found in TM-b (R_643_K_644_S_645_) in the equivalent neighborhood (data not shown). The full peptide sequences of zebrafish TM-a and TM-b were subjected to on-line prediction for 3D structure. The results showed striking similarity between human TM and zebrafish TM-b with the potential ezrin/actin binding site located in a comparable position (Fig. 1e). These results suggested that both zebrafish TM-like proteins, especially zebrafish TM-b, are structurally similar to human TM. The structural resemblance of TM among species also suggests the evolutionary conservation of TM.

### Altered expression of zebrafish TM-b resulted in embryogenic and hemostatic anomalies

**Fig. 2:**
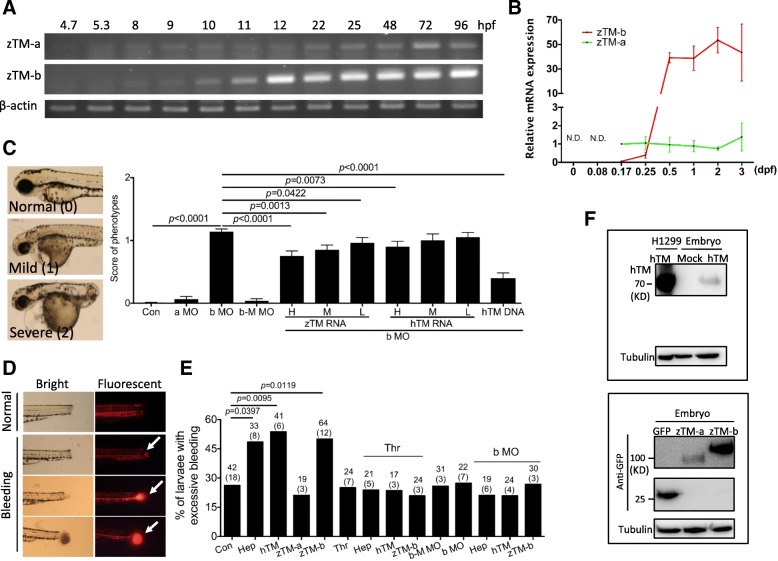
Zebrafish TM-b is functionally comparable to human thrombomodulin. Stage-dependent expression of zebrafish TM-like transcripts was examined by RT-PCR (**a**) and real-time PCR (**b**) with the cDNA prepared from the embryos of indicated stages. The relative mRNA expression levels were normalized with the mRNA levels of zebrafish β-actin and zTM-a at 0.17 dpf embryos by using 2^-△△Ct^ method. **c** Zebrafish embryos were injected with MO specific to zebrafish TM-a (a MO, 0.92 pmol/embryo) or TM-b (b MO, 0.46 pmol/embryo) with/without co-injecting the plasmids expressing recombinant human TM (100 pg/embryo) or cRNA of zebrafish TM-b or human TM (H: 2130.8 pg/embryo; M: 1733.1 pg/embryo; L: 1155.4 pg/embryo) at 1 to 2-cell stages. Embryos injected with TM-b MO with five mismatched nucleotides (zTM-b mismatched MO) were used as the control for comparison. Larvae were examined at 3 dpf for survival and categorized into normal, mild and severe abnormal based on their gross morphology (left) and quantified (right). The categorization for mild and severe phenotypes was mainly based on the severity of heart edema and extent of trunk development: embryos displayed observable pericardial edema and body curvature were categorized as mild; whereas those displaying apparent pericardial edema and dorsalization were categorized as severe. **d**, **e** Control or experimental larvae at 3 dpf were injected with the indicated tested reagents with Texas red at common cardinal vein (CCV). Larvae were incubated at 28 °C for 30 min and amputated to remove tails before recording for their hemostatic activity. Larva displaying excessive blood leakage with more apparent diffusion of blood/Texas red from the wound, as compared with control larvae, was categorized as bleeding phenotype and recorded. The numbers on top of each column represent the total number of larvae tested (without parenthesis) in the total number of repeats (with parenthesis). **f** Embryos injected with plasmids encoding either human TM, zTM-a or zTM-b at 1 to 2-cell stages were harvested at 24 hpf and immunoblotting for the expression of recombinant TMs with anti-hTM antibodies (left) and anti-eGFP antibodies (right). The extracts of H1299 cells transfected with plasmids expressing human TM were served as a positive control. The tested reagents included: heparin (Hep; 57.5 ng/larva), thrombin (Thr; 23 μU/larva), plasmids encoding zebrafish TM-a (zTM-a; peGFP N1-zTM-a, 200 pg/larva for hemostatic assay and 460 pg/larva for Western blotting), plasmids encoding zebrafish TM-b (zTM-b; peGFP N1-zTM-b, 200 pg/larva for hemostatic assay and 920 pg/larva for Western blotting), zTM-b MO (b MO), zTM-b mismatched MO (b-M MO), and plasmids encoding human TM (hTM; pcDNA3.1-hTM, 100 pg/larva for hemostatic assay and 800 pg/larva for Western blotting)

Analysis for the mRNA levels revealed that both TM-a and TM-b were expressed in the developing embryos at early stages. The RT-PCR signals were detected in embryos before 6 h post-fertilization (hpf) for both TM-a and TM-b (Fig. 2a). The band intensities became more distinguishable at 8 hpf and gradually enhanced as embryogenesis proceeded, especially for TM-b. Similar results were obtained for real-time PCR, where TM-a was steadily expressed throughout embryogenesis; whereas TM-b mRNA was increased continuously and significantly after 10 hpf (Fig. 2b). These results implied a role for zebrafish TM-like proteins in embryogenesis. Knocking-down TM-a with specific morpholino oligonucleotides (MO) caused no appreciable abnormality in larval gross morphology and survival (data not shown). On the other hand, knocking-down TM-b resulted in apparent anomalies in several tissues, including pericardial edema, smaller eyes and body curvature (Fig. 2c) The single exon of zebrafish TM-b gene structure and the unavailability of antibodies against zebrafish TM prevented us from using splicing site MO and immunoblotting to confirm the knockdown specificity and efficiency. Instead, the plasmids containing the nucleotide sequence of zebrafish TM-b MO target site fused with eGFP coding sequence (zTM-b-eGFP) was co-injected with either TM-b MO or control MO into embryos for examining the MO efficiency. Meanwhile, MO comprising five mismatched residues (zTM-b mis-matched MO) had also been injected in parallel as a negative control. The fluorescence intensity displayed by the embryos co-injected with TM-b MO was significantly weakened as compared to those with TM-b mismatched MO, supporting MO specificity (Additional file 1: Figure S2). Co-injecting zebrafish TM-b mRNA, human TM mRNA or the plasmids encoding human TM with TM-b MO effectively rescued the aforementioned anomalies, further confirming the knockdown specificity (Fig. 2c). Conversely, injecting the embryos with either hTM DNA or zTM-b mRNA alone caused no drastic change in larval gross morphology. Minor morphological anomalies, including curved tail and small head, were occasionally observed in less than 5% of the injected embryos (data not shown). These results support the essentialness of TM-b for embryonic development.

Excessive bleeding was observed in the larvae over-expressing zebrafish TM-b. The potential of zebrafish TM-a and TM-b in modulating hemostasis was examined since TM has long been known for its anti-coagulation activity in mammals. The activity of zTM-a and zTM-b expressed in HEK-293 T were examined in vitro with an activity assay in the presence of human protein-C and substrate peptide-nitroanilide diacetate. No significant protease activity, corresponding to the activated protein-C, was detected in the samples expressing zTM-a-eGFP and zTM-b-eGFP (Additional file 1: Figure S5). A protocol for evaluating larval hemostatic activity and zTMs anti-coagulability in vivo was also developed. The circulation of larvae at 3 dpf was visualized by ventricular injection with Texas-red dextran, with/without co-injecting the tested reagents. After a 30-min incubation at 28 °C, larvae were amputated to remove their tails and evaluated for the bleeding from the wounds under a fluorescence dissecting microscope. Excessive leakage of blood/Texas-red from the wound was considered bleeding (Fig. 2d). Injecting human thrombin (Thr), the key activator of coagulation, caused no apparent difference in the severity and occurrence of larval bleeding, as compared to control larvae (Fig. 2e). Injecting heparin (Hep), an anti-coagulant functioning by enhancing thrombin removal, significantly increased the bleeding of larvae. This heparin-induced increase in bleeding was abolished by co-injecting human thrombin, validating the reliability of this protocol. To examine the activity of zebrafish TM-like proteins in modulating hemostasis, embryos were injected with the plasmids encoding hTM or the plasmids encoding zebrafish TM-a or TM-b (zTM-a or zTM-b) fused with eGFP at 1-cell stages and raised to 3 dpf. Using human TM for the rescue would not only avoid morpholino binding but also provide the information on the functional conservation of TM during evolution. It also allowed us to confirm the expression of injected TM with Western blotting using the available anti-hTM antibodies (Fig. 2f). Excessive bleeding was observed in the larvae expressing either human TM or zebrafish TM-b, but not TM-a, which was alleviated by co-injecting thrombin subsequently (Fig. 2e). Knocking down zebrafish TM-b also relieved the excessive bleeding caused by heparin or increased expression of human TM. It should be noted that the phenotypes, including both larval morphology and bleeding phenomenon, displayed by those control embryos injected with scrambled MO and zTM-b MO containing five mismatched nucleotides (b-M MO) were comparable to those of wild-type (Fig. 2c and e). Together with the co-injecting experiments to demonstrate the knockdown efficiency and specificity of zTM-b MO (Additional file 1: Figure S2), our results showed that zebrafish TM-b modulated larval hemostasis and functionally resembled human TM.

### The distribution of zebrafish TM-b mRNA was spatiotemporally dependent during embryogenesis

**Fig. 3:**
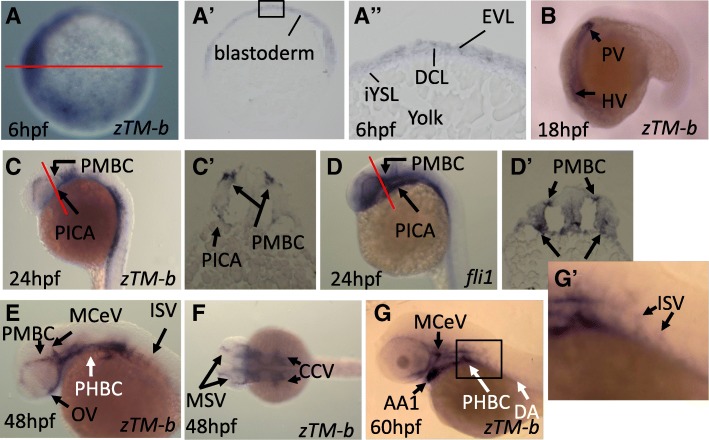
Spatiotemporal expression of zebrafish TM-b during early embryogenesis. Whole-mount in situ hybridization was performed using the embryos at the indicated stages with the riboprobes specific to zebrafish TM-b or endothelial marker for blood vessels (*fli1*). Embryos were imaged either from the animal pole (**a**), lateral view (**b**~**e** and **g**) or dorsal view (**f**). (A~A’’) The stained embryos were cross-sectioned sagittally (red line) and displayed in lateral view with animal pole to the top (A’). The higher magnitude of blastoderm in the squared area was also shown (A’’). Embryos of 24 hpf probed for the distribution of *zTM-b* (**c**) and *fli1* (**d**) were cross-sectioned along the plane indicated by red lines (C′ and D’). (G and G’) The image of embryo at 60 hpf was enlarged to show the ISVs signal. EVL: Envelop layer; DCL: Deep cell layer; iYSL: internal Yolk syncytial layer; HV: Head vasculature; PV: Presumptive vasculature; HBV: Head blood vessel; ISV: Intersegmental vessel; DA: Dorsal aorta; PCV, Posterior cardinal vein; PMBC, Primordial midbrain channel; PICA, Primitive internal carotid artery; OV, Optic vein; PHBC, Primordial hind-brain channel; MCeV, Mid-cerebral vein; MSV, Mesencephalic vein; AA1, Mandibular aortic arch

Characterization for the mRNA distribution in developing embryos showed that zebrafish TM-b expressed in a stage- and tissue-specific manner with prominence at vasculatures. The WISH signal corresponding to zebrafish TM-b transcripts was faint but observable in the blastoderm at 6 hpf (Fig. 3A-A’’). As embryogenesis proceeds, TM-b signal became recognizable in most presumptive vasculatures, including head vasculatures (HV), primitive internal carotid artery (PICA) and primordial midbrain channel (PMBC, the vessel between the anterior cerebral vein and the mid cerebral vein), which were overlapped with the mRNA signal for *fli1*, a vessel marker (Fig. 3B-D’). The expression of TM-b was apparent in most primordial and developed vessels in both head and trunk, including optic vein (OV), primordial hindbrain channel (PHBC), mid-cerebral vein (MCeV), mesencephalic vein (MSV) and mandibular aortic arch (AA1) by 48 hpf and also in dorsal aorta (DA) and intersegmental blood vessels (ISV) at 60 hpf (Fig. 3E-G’). These data supported the importance of zebrafish TM-b in vessel development and function in developing embryos.

### Zebrafish TM-b morphants displayed circulation defects

**Fig. 4:**
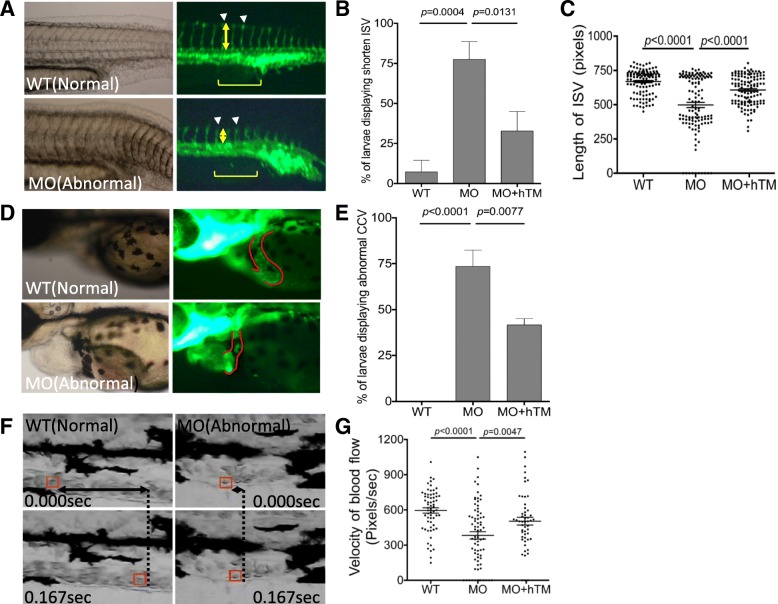
The impact of zTM-b knockdown to vessels formation. Tg (*fli 1*:eGFP) embryos were injected with TM-b MO with/without co-injecting plasmids encoding human TM, as described in Material and Methods, and observed for the development of ISV at 28 hpf (**a**-**c**) and CCV at 56 hpf (**d**, **e**). The percentage of morphants displaying anomaly in ISV (arrowheads) and CCV (CCV boundary circled by solid line) were also recorded. The extent of incomplete extension observed in ISV was quantified by calculating the length (yellow double-headed arrows) of five ISV singlets (brackets) immediately adjacent to cloaca for each morphant. **f**, **g** Larvae at 3 dpf were video-recorded laterally for the trunk area with anterior to the right for blood flow. A single red blood cell (boxed in red) inside the caudal vein was traced and calculated for its traveling distance (F; double-headed arrows) and velocity (**g**) by analyzing the serial video still frames within 0.167 s. Presented are the results collected from at least three independent experiments including total 16–35 larvae for each experimental group. WT, wild-type embryos; MO, zTM-b morphants; hTM, human thrombomodulin

Knocking-down zebrafish TM-b impeded larval circulation. Abnormal vessels development and obstructed circulation were observed in zebrafish TM-b morphants. The transgenic line Tg (*fli1*:eGFP) expresses eGFP driven by the vessel-specific promoter *fli1* and allows for a real-time observation for vessels formation [34]. In zebrafish, the development of common cardinal veins (CCV) is one major event of vasculogenesis; whereas the sprouting of intersegmental vessels (ISV) is a process of angiogenesis. The larvae displayed incomplete ISV with shortened extension at 28 hpf were categorized as abnormal, which were found in approximately 70% of Tg (*fli1*:eGFP) TM-b morphants (Fig. 4a~c). Abnormal CCV with smaller size and retarded curvature was also apparent at 56 hpf in 80% of Tg (*fli1*:eGFP) embryos with TM-b knockdown (Fig. 4d and e). The flow rates of larval blood stream were calculated from the traveling distance of individual red blood cells captured in a series of video stills. Abnormal flow rate with significant decrease was also observed in zebrafish TM-b morphants (Fig. 4f, g, Additional file 2: Movie S1 and Additional file 3: Movie S2). Co-expression of human TM significantly rescued the aberrant development of vasculature and defective circulation in TM-b morphants, confirming the knockdown specificity and contribution of TM-b to the development and function of vessels.

**Fig. 5:**
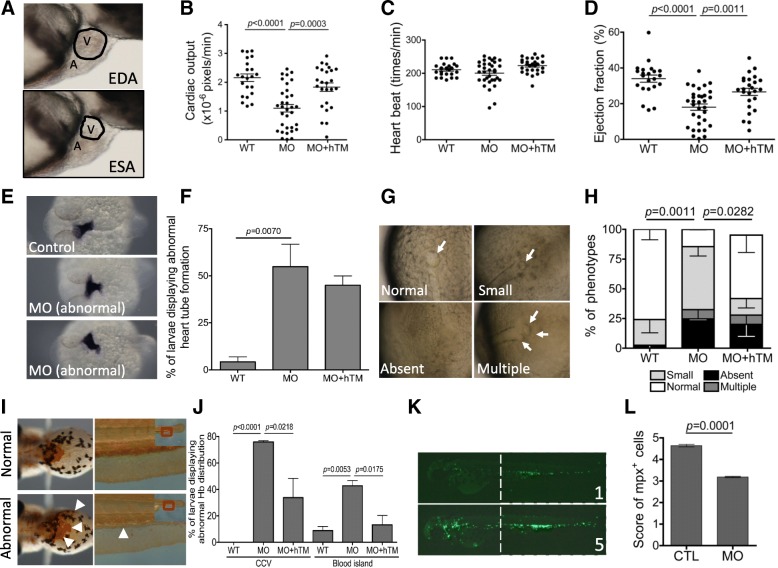
The impact of zTM-b knockdown in heart and blood cell formation. Wild-type and Tg (*mpx*:eGFP) embryos were injected with TM-b MO with/without co-injecting plasmids encoding human TM and examined for the development of heart and blood cells. **a**-**d** The cardiac ejection fraction and cardiac output of larvae at 3 dpf were calculated from the change in the dimension of cardiac chamber (the circled area in (**a**)) in the serial video still images (lateral view with anterior to the right) as described in Materials and Methods. **e**, **f** Embryos at 1 dpf were subjected to WISH for the distribution of *cmlc2* transcripts, a cardiac primordium marker. Embryos displaying signal with decreased intensity or spatially aberrant distribution (heart tube with mid-line positioning or right-jogging) were categorized as abnormal and quantified. **g**, **h** The development of embryonic right-left patterning was evaluated at 12 hpf and categorized into normal, small, absent and multiple based on the number and morphology of Kupffer’s vesicle (arrows). **i**, **j** Embryos at 3 dpf were stained with o’-dianisidine for hemoglobin distribution. Larvae displaying weaker, dispersed or ectopic signals in common cardinal veins (arrowheads in left panel) and/or absence of posterior blood island (arrowheads in the right panel) were categorized as abnormal and quantified. **k**, **l** Tg (*mpx*:eGFP) embryos with/without TM-b knockdown were imaged at 2 dpf and scored from 1 to 5 points based on the intensity of green fluorescence, which represented the quantity of *mpx*-positive leukocytes (mainly neutrophils). Data were collected from at least 3 independent experiments with the total embryo numbers of 11–84 for each group. WT, un-injected wild-type embryos; MO, embryos injected with TM-b MO; hTM, human thrombomodulin; CTL, Tg (*mpx*:eGFP) embryos without MO injection; A, atrium; V, ventricle; EDA, end-diastolic area; ESA, end-systolic area

In addition to the aforementioned aberrant heart morphology and edema, cardiac parameters were affected in zebrafish TM-b morphants. Larval cardiac ejection fraction and output were estimated from the difference between diastolic and systolic cardiac areas via the images analysis on serial video stills as described in Materials and Methods. TM-b morphants displayed an approximately 50% decrease in both ejection fraction and cardiac output. No apparent change was recorded for the averaged heart rate, as compared to that of wild-type (Fig. 5a~d). The WISH results with riboprobes specific to the cardiac marker *cmlc2* showed aberrant signal distribution of heart primordium in TM-b morphants (Fig. 5e and f). We observed approximately 24.6 and 26.2% of TM-b morphants displaying right-jogging and middle line-positioned cardiac tube, respectively, suggesting uncoupling of D-looping (the subsequent repositioning of the ventricle to the right of the atrium). The presence of incorrectly, and sometimes oppositely, positioned cardiac primordium implied a disturbance in the establishment of left-right asymmetry during embryogenesis. These results prompted us to examine the development of Kupffer’s vesicle, a ciliated organ of asymmetry which is transitorily formed at the end of gastrulation in the tail region of developing teleost embryos and initiates left-right development of the brain, heart and gut. Aberrant formation of Kupffer’s vesicle, including absence and presence of multiplex, was observed in zebrafish TM-b morphants, suggesting interfered left-right patterning in morphants (Fig. 5g and h). The development and distribution of red blood cells were characterized by O′-dianisidine staining for hemoglobin. Dispersion of hemoglobin signal in the CCV region was observed in 3 dpf morphants (Fig. 5i and j). In addition, the lack of clearly observable blood island was found in approximately 50% of zTM-b morphants. Both were successfully rescued by co-injecting hTM mRNA. Knocking down zebrafish TM-b in Tg (*mpx*:eGFP) embryos also significantly reduced the number of cells glowing with green fluorescence (Fig. 5k and l). Tg (*mpx*:eGFP) is a transgenic line expressing eGFP specifically in neutrophils [46]. Normally, abundant green fluorescent signal, representing the normal distribution of neutrophils, is apparent in control Tg (*mpx*:eGFP) larva, as shown in the lower panel of Fig. 5k (scored 5); whereas knocking-down zTM-b diminished neutrophils to varied extent. Shown in the upper panel is an example representing the morphant with significant decrease in the number of neutrophils (scored 1). Most larvae in morphants group displayed the morphological abnormalities with the severity ranging between score 1 and score 5. These results suggested that decreasing zebrafish TM-b expression also interfered with hematopoiesis. Nevertheless, all the above abnormalities, including both heart and blood cells formation, were rescued by co-expressing human TM. Together with the anomalies observed in the heart, blood-flow and vessels of TM-b morphants, our results supported that TM-b was crucial to the development of zebrafish circulation system.

### Zebrafish TM-b morphants displayed aberrant neural and sensory tissues development

**Fig. 6:**
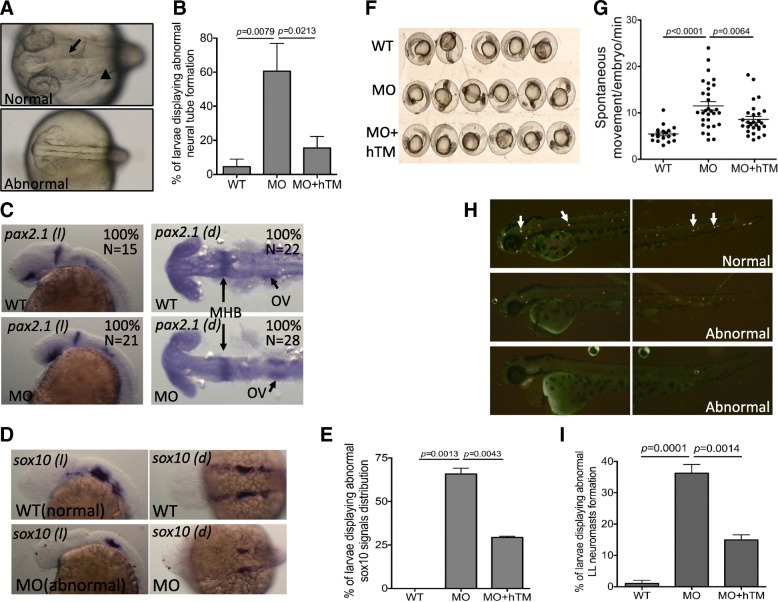
The impact of zTM-b knockdown in neural development. Wild-type embryos, subjected to with/without injecting TM-b MO and rescuing with hTM, were observed and collected at the indicated stages for evaluating the neural tissues development. **a**, **b** Embryos at 1 dpf were imaged dorsally under a light dissecting microscope. Those embryos displaying absence or aberrant formation of brain ventricle (arrow) and brain structure (such as mid-hind brain boundary indicated by arrowhead) were categorized as abnormal and quantified. **c**-**e** Embryos at 1 dpf were subjected to WISH for the distribution of *pax2.1* (a marker of central nervous system) and *sox10* (a marker of neural crest cells). Embryos were imaged both laterally (*l*) and dorsally (*d*) with anterior to the left. **e** Embryos displaying signal with altered intensity or distribution pattern for *sox10*, in comparison to wild-type, were categorized as abnormal and quantified. **f**, **g** Larval spontaneous movement was examined by video-recording the embryos at 1 dpf for 5 min and counting for the numbers of moving episode for each embryo. Presented here is a representative image of a series of video stills showing the arrangement of examined embryos. **h**, **i** The neuromasts of lateral line (arrows) in embryos of 2 dpf were visualized by staining with 4-Di-2-Asp and imaged laterally with anterior to the left. Those embryos displaying absent or aberrantly distributed neuromasts were categorized as abnormal and quantified. Data were collected from at least 3 independent experiments with the total sample numbers of 18–127 for each group in different experiments. WT, wild-type embryos; MO, zTM-b morphants; hTM, human thrombomodulin; OV, Otic vesicles

Abnormal neural development with brain ventricles malformation was observed in zebrafish TM-b morphants at 24 hpf. The smaller head observed in TM-b morphants prompted us to hypothesize that knocking down TM-b impeded neural tissues formation. Unlike the wild-type control embryos, no distinctive cranial cavity and mid-hindbrain boundary were formed in the central region of forebrain and midbrain in TM-b morphants (Fig. 6a and b). The WISH signal reflecting the development of mid-hindbrain boundary and spinal cord (*pax2a*) did not show significant difference between wild-type controls and TM-b morphants (Fig. 6c). Nevertheless, aberrant distribution of *sox10*, a neural crest cells marker, was observed in the larvae knocked-down for TM-b (Fig. 6d and e). Neural crest cells are the common precursors of a wide spectrum of cells, including neural and sensory tissues. Zebrafish TM-b morphants of 24 hpf also displayed significantly increased frequency of spontaneous movement, a reflection of locomotor networks (Fig. 6f, g and Additional file 4: Movie S3). Incomplete development or completely absence of lateral lines was observed occasionally when TM-b morphants reached 48 hpf (Fig. 6h and i). The *lateral line* is a *system* of sense organs found in teleost fish and amphibians and used to detect movement, vibration, and pressure gradients in the surrounding water. It comprises a set of regularly spaced neuromasts arranged in line on the body surface of the fish and is closely related to the mammalian auditory system. During embryogenesis, the primordial cells originating from cephalic placodes at the anterior head region proliferate, migrate and are deposited along the horizontal myoseptum to the tip of the tail in the manner resembling collective cell migration, forming the neuromasts of lateral line [47, 48]. We found that the neuromasts in both anterior and posterior trunk of TM-b morphants were significantly decreased in number and sometimes even completely absent. Co-expressing human TM effectively prevented against the occurrence of all the above defects described for TM-b morphants, supporting the specificity and causal effect of TM-b knockdown. Our results showed that TM-b contributed to the development and function of the central and sensory neural systems in zebrafish.

### Zebrafish TM-b promoted cohesive movement of germ layers during epiboly

**Fig. 7:**
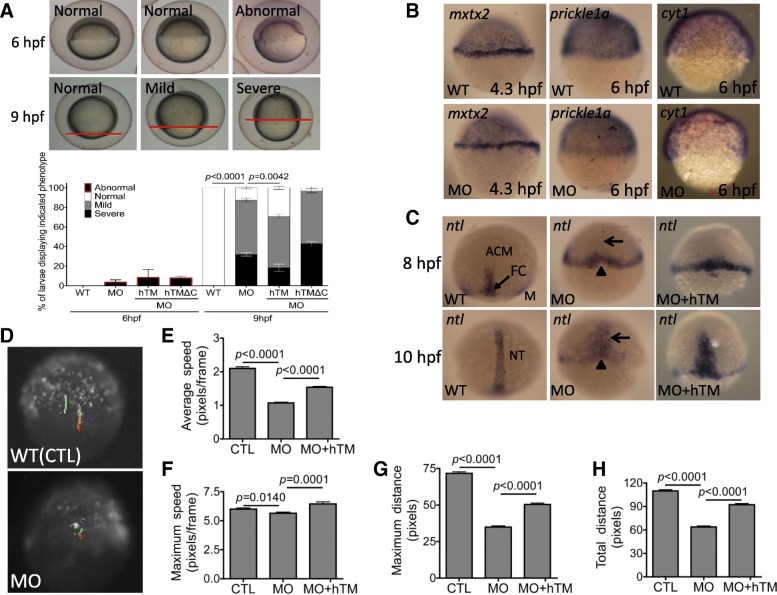
Knocking-down zTM-b impeded embryonic cells migration. Embryos of both wild-type and zTM-b morphants were monitored for epiboly progression during early embryogenesis. **a** The epibolic abnormality was categorized based on the extent of blastoderm movement. The progression of epiboly recorded at 6 hpf was categorized as abnormal when the extent of epiboly was less than 50%. The progression of epiboly recorded at 9 hpf was categorized as normal, mild delay (between 75 and 90% epiboly) and severe delay (less than 75% epiboly) based on the extent of epiboly (upper panel) and quantified (lower panel). The embryos were also co-injected with plasmids encoding human TM or human TM with truncated intracellular domain for rescue. The amino acid residues from 523 K to 557 L in human TM were removed in the hTM truncated construct TMdelC-pEGFP/N1. **b** The progression of epiboly in wild-type embryos and zTM-b morphants with/without hTM rescue were characterized by WISH with the riboprobes specific to margin and YSL (*mxtx2*), marginal epiblasts (*prickle1a*) and EVL (*cyt1*)*,* respectively, at the indicated stages. **c** Convergent extension movements of epibolic blastomeres were examined by WISH with *ntl*-specific riboprobes. Lack of forerunner cells (FC) at 8 hpf and shortened and broadened notochord (NT) at 10 hpf were apparent in zTM-b morphants. **d** Both wild-type embryos and zTM-b morphants at 64-cell stage were injected with plasmids expressing eGFP (peGFP N1, 150 pg/embryo) into one single cell and continuously traced under a fluorescence dissecting microscope from 6 hpf to 7 hpf for the migration of the injected cells. The migratory parameters of the recorded cells, including average speed (**e**), maximum speed (**f**), maximum distance (**g**) and total distance (**h**), were calculated with the on-line software CellTracker on MATLAB R2015a system. Reported are the averages calculated from total of 11 cells selected from three different embryos for each group. All images were shown in lateral view with animal pole to the top. WT, un-injected wild-type embryos; MO, zTM-b morphants; hTM, human thrombomodulin; hTM△C, human thrombomodulin with truncated-intracellular domain

The wide-spectrum of anomalies observed in TM-b morphants and the aberrant development of lateral line prompted us to hypothesize that these phenotypes could be secondary to the primary defects in cell migration during epiboly and early embryogenesis. Epiboly is a process occurring during early embryogenesis, which involves massive movement of blastomeres in the manner of coordinated cells migration and allows for dramatic physical restructuring. Detailed recording for epiboly progression revealed epiboly delay in TM-b morphants at 9 hpf but not in morphants before 6 hpf (Fig. 7a). Co-injecting human TM significantly rescued epiboly delay. Interestingly, in contrast to the full-length TM, co-injecting the TM lacking intracellular domain did not rescue the delayed epiboly observed at 9 hpf in zTM-b morphants. These results indicated the essentialness of intracellular domain for TM to be functional in promoting epiboly in which interaction and participation of both actin and microtube are essential. The distribution and intensity of WISH signals marking YSL (mxtx2), epibolic margin (prickle 1a) and envelop layer (cyt1) were comparable between wild-type control embryos and TM-b morphants before 6 hpf (Fig. 7b). The progression of epiboly involves extensive cell extension and intercalation, besides the massive cell migration, which occur mainly in deep cells layer (DEL) of blastoderm. Assessing epiboly progression by probing DEL cells with riboprobes *ntl* (no tail), a pan-mesodermal marker for blastomeres intercalation during gastrulation, revealed epibolic anomalies in TM-b morphants. A shortened axial chordal mesoderm and broadened notochord were found in morphants at 8 hpf and 10 hpf (Fig. 7c). All the above migratory anomalies observed in the blastomeres of TM-b morphants were prevented by co-expressing human TM.

Characterization for the movement of single cell in blastoderm showed that knocking-down TM-b impeded the migration of individual blastomere in early embryos. The migration of individual blastomeres in the epibolic blastoderm of WT or TM-b morphants at 64-cell stage was traced by the strategy of single-cell labeling, in which a single blastomere was injected with plasmids encoding GFP. Results showed that all cell migratory parameters, including averaged speed, maximum speed, maximum distance and total distances, were significantly decreased with no apparent directional bias in the blastomeres of TM-b morphants (Fig. 7d~h). These results suggested that TM-b was required for the embryonic epithelial cell intercalation and collective movement at very early stages during embryogenesis.

**Fig. 8:**
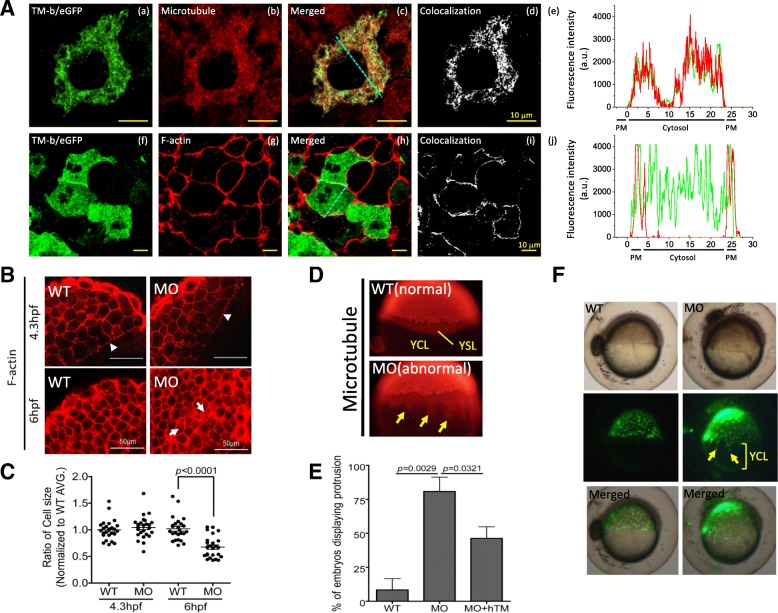
Cellular localization of zTM-b in developing embryos. **a** Wild-type embryos injected with plasmid zTM-b fused with eGFP (a, f; peGFP N1-zTM-b, 200 pg/embryo) at 1-cell stage were stained for microtubule (b) and F-actin (g) at 6 hpf with anti-α-tubulin antibodies and Alexa Flour 594 Phalloidin, respectively. (a-c, f-h) Confocal microscopy revealed distribution and colocalization of these proteins under excitation with a 488, or 543 nm laser. Blue dashed lines indicate the area of quantitative analyses of TM-b/microtubule/F-actin fluorescent intensity shown in (e) and (j). (d, i) Whole-cell pixel-by-pixel analyses of TM-b and microtubule or TM-b and F-actin colocalization. Scale bars, 10 μm. (e, j) Quantitative fluorescent analyses of cellular TM-b/microtubule/F-actin distribution. PM: plasma membrane. **b**, **c** Cell size was estimated from the dimension of blastomeres in the confocal images of phalloidin 594-stained embryos (**b**). Actin signal was detected in cell peripheral and actin ring at the front edge of epibolic blastoderm (arrowheads). Increased signal dispersed in cytoplasm was also observed (arrows). For calculating cell dimension, total of 25 cells selected from 5 images for each group were analyzed with the on-line software Image J. Reported are the relative cell size normalized with the averaged size of wild-type blastomeres of the same stages. **d**, **e** Wild-type embryos injected with TM-b MO, with/without hTM rescue, were immuno-stained with anti-α-tubulin antibodies at 4.3 hpf. Embryos displaying signal with spike-like protrusion into YCL (arrows) were observed in zTM-b knockdown group and quantified. **f** Embryos at 4 hpf were injected with SYTOX green (0.25 mM/2.3 nL/embryo), a vital dye specific for nucleus staining, at the junction of blastoderm and yolk. The injected embryos were observed continuously under a dissecting fluorescence microscope and imaged at 6 hpf. Some nucleuses (arrow) within YSL moved ahead of the margin of blastoderm in TM-b morphants. Images were taken from lateral view with the animal pole to the top. YCL, yolk cytoplasmic layer; YSL, yolk syncytial layer; WT, wild-type embryos; MO, zTM-b morphants; hTM, human thrombomodulin

Further characterization revealed the co-localization of TM-b with cytoskeleton and modulated the cohesive movement of yolk syncytial layer (YSL) in developing embryos. Cytoskeleton is crucial in modulating cell migration and embryonic epiboly. To examine the cellular localization of TM-b in a living embryo, the plasmids encoding TM-b-eGFP recombinant protein with signal peptide were injected into embryos at 1 cell-stage and observed for the presence of green fluorescence at 4.3 hpf. The results showed that the fluorescence signal corresponding to TM-b in blastomeres was distributed in cellular cortex and cytoplasm and co-localized with both F-actin and microtubule, respectively (Fig. 8a). In addition, the amount of TM-b that co-localized with microtubule and F-actin were 78.77 and 11.95%, respectively (Fig. 8Ad, Ai). Knocking-down TM-b caused no obvious change in the gross morphology of blastomeres at both 4.3 hpf and 6 hpf, but apparently increased the intracellular puncta distribution and dispersion of actin signal in cytosol (Fig. 8b). Significant decrease in the cell size at the two-dimensional view of blastomeres was also observed (Fig. 8c). Staining for microtubule revealed desynchronization of moving blastodermal layers in TM-b morphants during epiboly. Unlike in control embryos, TM-b morphants displayed irregular protrusion into yolk cytoplasm at the margin of the moving blastoderm (Fig. 8d). This protrusive phenomenon was significantly prevented by co-expression of human TM (Fig. 8e). To distinguish the identity of the protrusion among the multilayers of blastoderm, SYTOX green, a vital dye specific for nucleic acid and capable of quickly staining the nuclei in YSL, was injected into embryos at the interface between yolk and blastoderm at 4 hpf. The quick spreading of green fluorescence detected in the nuclei below the epiblast boundary indicated the uncoupling of YSL in TM-b knockdown embryos (Fig. 8f). These results suggest a role for TM-b in modulating the cohesive movement of YSL within the multilayered epithelium.

## Discussion

TM is an integral membrane protein well-known for its conventional anticoagulant activity. In the current study, we report the structural and functional comparability between mammalian TM and zebrafish TM-b. We showed that zebrafish TM-b possesses anti-hemostatic activity and is expressed mainly in vasculature. In addition, TM-b is important for germ layer formation and organogenesis. Our results showed that besides actins, TM-b also interacted with microtubules. The significance of TM-b co-localization with cytoskeleton in blastomeres and YSL is reflected by the impeded epibolic progression observed in TM-b morphants, substantiating the potential of TM-b in modulating collective cell migration in vivo. The elucidation of structural and functional conservation between human TM and zebrafish TM-b also supported the properness of using zebrafish as an in vivo platform for studying the biological significance and medical use of TM.

Zebrafish TM-b interacts with both actins and microtubules and modulates the movement of blastomeres in germ layers. We observed epiboly delay in zebrafish TM-b morphants. Epiboly is the earliest morphogenetic movement and a very crucial stage during embryogenesis. It involves coordinated cell shape changes and expansion of cell layers, which allow for the development of tissue architecture. Completion of epiboly requires the coordination of cytoskeletal dynamics across the embryo. During epiboly, the ring-like structures of filamentous actin at the leading edge of enveloping layer (EVL) retains the external yolk syncytial nuclear (YSN) within the margin of EVL. The actin bundles progressively formed in the vegetal cortex of yolk cell provide enormous force and act in concert with marginal actin ring to pull external YSN toward vegetal pole [49]. The progression of epiboly also heavily depends on the organization and function of two distinct microtubule arrays located in the cortical cytoplasm of yolk cell: one originating from the marginal blastomeres and intercrossing to form microtubule network in YSL; the other emanating from the microtubule network of the syncytial layer and extending from animal pole to vegetal pole in yolk cytoplasmic layer (YCL). The changes in the organization of these microtubule arrays during epiboly tightly correlate with germ layers formation since disrupting microtubule depolymerizing completely blocked the epibolic movements of the YSN and led to the incoherent movement of YSL [50]. Our observations that zebrafish TM-b co-localized with cytoskeleton, including both actin and microtubule, support the involvement of TM-b in modulating epibolic progression via interacting with cytoskeleton. In addition, the involvement of TM-b in blastomeres migration during germ layer formation provides an explanation for the widespread defects observed in TM-b morphants which did not seem to match the expression domains of TM-b in larvae at later stages.

The loss of cohesive movement of YSN/YSL in zebrafish TM-b morphants further supports the concept of the functional and concomitant association between zebrafish TM-b and microtubule in coordinating epiboly. The irregular protrusion of microtubule bulging into YCL and ahead of the EVL rim indicated a dissociation between YSL and the migratory deep cells (DEL). Our data suggest that knocking-down zebrafish TM-b interrupted F-actin integrity, thereby impairing epiboly. The increased intracellular puncta distribution and dispersion of actin signal observed in the cytosol of blastomeres indicate that zebrafish TM-b partakes in actin recruitment and contributes to the synchronic movement of the blastodermal multi-layers by connecting the blastomeres and the moving of YSL along the animal-vegetal axis during epiboly. These observations implied the possibility that knocking-down TM-b might interrupt intracellular actin dynamics and echoes our previous findings that human TM is linked to actin cytoskeleton via ezrin and takes part in the formation of E-cadherin-based adherens junctions [23]. It was likely that knocking-down zebrafish TM-b disrupted actin-associated complex in blastomeres and disturbed the connecting force among blastomeres, as well as the coordination between the moving blastodermal layers. This speculation was further supported by the subsequent observation that blastomeres with zebrafish TM-b knockdown displayed retarded migratory activity in the epibolic blastoderm. In addition, the smaller blastodermal cells observed in TM-b morphants provide additional evidence echoing the potential interplay between TM-b and actin since the actin cytoskeleton has been shown to involve in cell volume regulation by sensing and mediating extracellular signals [51]. Meanwhile, actin cytoskeleton-associated proteins were also reported to participate in cell volume regulatory response [52], further supporting the role of TM-b in modulating cell volume in collaborating with actin. How zebrafish TM-b contributes to the actin-microtubule crosstalk and functions in concert with both actin and microtubule simultaneously in modulating collective migration warrant further investigation.

The development of both CCV and ISV was disrupted when zebrafish TM-b was knocked-down (Fig. 4a-e), evidencing the importance and conservation of zebrafish TM-b in endothelium and vasculogenesis/angiogenesis. In zebrafish, the first embryonic primary vasculature, including the dorsal aorta, cardinal vein, and primitive cranial vessels, is formed by vasculogenesis. Following the primary vasculogenesis is the angiogenesis for most subsequent vessels formation [31]. The migration of the sprouts developed during angiogenesis is dynamic with numerous filopodia-like cellular processes extending from the tips of the growing sprouts in all directions. We had shown in our previous studies that TM interacted with α-actinin-4 and ezrin, which link to actin filaments, and affected cell morphology by modulating cytoskeleton formation and regulating cellular lamellipodia protrusion, spreading and stiffness [53]. Zebrafish TM-b appears to be strongly expressed in most vessels, especially those primitive endothelial channel and primordial vessels in developing embryos. Our previous study showed that ETS-1 mediates the transcriptional regulation of human TM expression [54]. ETS-1 is also a key regulator of vasculogenesis in zebrafish [55]. In addition, multiple ETS-1 binding sites are predicted in the promoter region of zebrafish TM-b, echoing the potential involvement of TM-b in vessel development (Additional file 1: Figure S3).

That knocking-down TM-b impeded heart development, hematopoiesis and neurogenesis is unexpected since TM-b expression in embryonic tissues other than vasculature was not impressive. Nevertheless, the potential of TM involving in the development of tissues other than vessels has not been addressed before. Several lines of evidences revealed in the current study and previous reports support a role for TM/TM-b in organogenesis. First, the malformed tissues in TM-b morphants were successfully rescued by co-expressing TM/TM-b, supporting the essentialness of this molecule for organs development. Second, cells rearranging and moving collectively in large-scale are the hallmarks of tissue remodeling and fundamental to embryonic morphogenesis. The impeded tissues formation observed in TM-b morphants is likely a consequence of obstructed cell migration and germ layers development due to TM-b knockdown. In addition, proper organogenesis *depends* on *cell shape changes and stiffness governed by mechanical forces and tensions, which is largely affected by actin filaments* [56–58]. TM had been shown to link to actin filaments and regulate cellular lamellipodia protrusion, spreading and stiffness [53]. One might argue that the observed anomalies in zTM-b morphants might merely result from a general growth delay. The knockdown of zTM-b indeed caused developmental delay in the very early embryogenesis, especially epiboly (Fig. 7). However, as the embryogenesis proceeds, the development of certain tissues has caught up and displayed the morphological characteristics that are comparable to the control embryos of the same stages. For instance, the patterns of midbrain-hindbrain boundary and the development of optic stalk (OS) and otic vesicle precursors (OV) (characterized by WISH for *pax2.1)* in zTM-b morphants are largely unchanged, suggesting that the embryos had developed following the timeline of embryogenesis (Fig. 6c). In addition, the abnormalities observed in some other tissues are apparently anomalous, instead of delay, since the aberrations remained till the later stages. Examples are the irregular shape of Kupffer’s vesicle, dysfunctional heart and decreased numbers of blood cells (Fig. 5). Therefore, the observed anomalies in zTM-b morphants could not merely result from a general developmental delay. This is further supported by the observation that abnormalities, including dorsalization, shorten trunk and the absence of inflated swim bladder, remain in zTM-b morphants even at 7 dpf (Additional file 1: Figure S4). As aforementioned, we could not completely exclude the possibility that some of the observed defects were actually the consequence of developmental delay occurring to certain primordial tissues, such as somite and ventricles, at early stage (Figs. 4 and 6). Our results at least imply a desynchronized organogenesis in zTM-b morphants. It should be noted that no apparent abnormality was reported for the blood vessels and other organs in TM-null mouse embryos before E15 although a loss of TM function causes embryonic lethality [11, 12, 14]. One of the origins for this inconsistency is the difference between animal models and the strategies employed to establish TM/TM-b deficiency and to rescue. The often-seen compensatory effects and the decreased survival rate of embryos at early stages due to the depletion of a gene function in mammalian models shall also be considered. Investigation on the tissue specific activity and functional mechanism of TM shall be valuable invaluable for further unveiling the contribution of TM to organogenesis.

The loss of mobility for blastomeres devoid of TM-b expression revealed the essentialness of TM in mediating epiboly and implies a passive movement for the collectively migrating blastomeres within the epibolic blastodermal germ layers. These results echo the notion that the migration of the neuromasts during posterior lateral line (pLL) formation is promoted by the leader cells at the leading edge of the collectively migrating pLL primordium [59]. It should be noted that the loss of mobility observed for epibolic blastomeres due to TM-b knockdown appears to be different from our previous observation that knocking down TM in A431 accelerated cell migration due to the loss of collective cell migration [23]. These differences most likely reflect the divergence among the two experimental platforms: epibolic blastomeres in developing embryos in vivo versus cultured stable cell-line in vitro, although the potential contribution raised from the intrinsic differences among species cannot be ruled out. The discrepancy in the complexity of the extracellular microenvironment, in which the targeted cells dwelled, is enormous between a developing embryo and the medium in a culture flask. The physiological status and context-dependence (proliferating and differentiating embryonic cells during organogenesis vs. proliferating stable cultured cells) of the targeted cells are also crucial determinants to cell mobility. These results added further emphasis to the importance of studies with in vivo platform. Further investigation on the mechanisms underlying these differences shall help elucidate the conserved biological functions of TM/TM-b in evolution and in diseases pathogenic processes.

In the current study, we have developed a protocol for examining the hemostatic activity of zTM-b in vivo by monitoring the extent of larval bleeding upon tail amputation. It should be noted that despite the numerous advantages of using zebrafish for biological research in many fields, evaluating the coagulability status of larvae with conventional hematology index, ie. activated prothrombin time (APTT) and prothrombin time (PT), is impractical due to the limited amount of blood that can be collected from a larva (approximately 1 μl). This disadvantage had prompted us to develop the in vivo assay for examining the larval coagulability and hemostatic activity of zTM-b. Intriguingly, we found that the anti-coagulability of zTM-b could only be detected with the in vivo protocol but not the in vitro assay in cultured cells. This difference may reflect the significant dissimilarity between the microenvironment complexity and the necessary interaction among molecules or cells lineages provided by an in vitro system and those by an in vivo platform. This discrepancy also provides a probable explanation for why the results obtained in vitro would sometime fail to recapitulate the observation in vivo. There are several possibilities that might contribute to the unsuccessfulness in detecting the anti-coagulability of zebrafish TM-b with in vitro assay. First, the likely structural variation between human and the plausible zebrafish Protein C may prevent the zTM-b/thrombin complex from acting on the targeted human Protein C. The activation of Protein C by interacting with thrombin/TM complex is necessary for measuring TM anti-coagulability in vitro. The Protein C used here is from human; whereas the gene/molecule analogic to human Protein C has not been identified in zebrafish to date. Therefore, the structural and functional comparability between human and zebrafish Protein C, if it did exist, remains unknown. It was likely that the potential incomparability in their structures between human and the plausible zebrafish Protein C might prevent the zTM-b/thrombin complex from interacting with human Protein C. Another possibility is that zTM-b may not mediate its anti-coagulation activity observed in larvae via the conventional APC-dependent pathway. Studies reported that thrombin/TM complex retains less than 1% of the fibrinogen clotting activity of free thrombin [60]. Similarly, the overexpressed zTM-b in embryos injected with zTM-b mRNA might significantly decrease the enzymatic activity of thrombin by merely binding and forming complex with thrombin, inhibiting coagulation. Again, the disagreement between zTM-b activity obtained from the assay performed in vitro and the biological function observed in vivo revealed the discrepancy between in vitro and in vivo environment. One shall be vigilant while conducting and comparing the in vitro and in vivo studies.

The reason that we preferentially employed the knockdown, instead of the knockout, strategy in the current study to unveil the biological function and evolutionary conservation of TM-b in zebrafish are two-fold. First, this is mainly for avoiding the potential ambiguity and difficulty in deciphering the function of TM-b due to the genetic compensation, a response occurring only in gene knockout animal but not in knockdown. Gene compensation is a response in which the activation/inactivation of other genes in a compensatory network allows the organisms to buffer against deleterious mutations and maintain cells/organism wellness [61]. This compensatory effect could lead to an apparently normal or “secondary” phenotype reflecting the compensatory effect but not the loss of the interested gene [62]. The occurrence of gene compensation will result in ambiguity and increase the difficulty in deciphering the biological significance of the interested gene. In fact, genetic compensation is a wide-spread phenomenon and has been documented in a number of model organisms ranging from fruit fly to mammal, including zebrafish [63]. Second, previous studies showed that the heterozygous TM deficient (TM^+/−^) mice appeared normal and were free of thrombotic complications; whereas the homozygous TM deficient (TM^−/−^) embryos died before embryonic day 9.5 [12]. Any occurrence of the aforementioned situations would render further investigation infeasible on the premise that they could also possibly occur in zebrafish knockout line. Considering the lack of information on zebrafish TM or TM-like molecule to date and to avoid the ambiguity and difficulty in deciphering the impact of decreased TM-b expression, we considered the knockdown approach is appropriate for our initial attempt to get a glimpse on the impact of decreased TM-b expression in embryogenesis. The results on the existence and fundamental characterization of zTM-b reported in the current studies would serve as a reference for the subsequent studies in depth, including the establishment of Crisper-Cas9 knockout fish underway and also a complementary comparison to those from knockout rodents.

## Conclusion

Our study identify a zebrafish TM-like molecule displaying structural and functional compatibility to mammalian TM. Our results suggest that this TM-like protein coordinates massive cells migration during epibolic progression via interacting with cytoskeleton and is crucial to the cohesive movement of blastodermal layers. Our results also support the properness of using zebrafish embryos as an in vivo platform for studying the interplay between TM and cytoskeletal dynamics/function and for investigating the significance of TM in EMT, a crucial process in tissue formation and cancer metastasis.

## Additional files

Additional file 1: **Figure S1.** The alignment and identity of zTHBD primary sequence with those of zebrafish TM-a and TM-b. **Figure S2.** Efficiency of zTM-b MO. **Figure S3.** Schematic diagram of the predictive ETS-1 binding sites in TM promoter. **Figure S4.** The zTM-b morphants did not recover from the developmental anomalies as the embryogenesis proceeding to the later stages. **Figure S5.** In vitro activity assay for thrombomodulin. (PDF 1830 kb)

Additional file 2: **Movie S1.** The video clip showing the traveling of red blood cells in wild-type larva. (MOV 1480 kb)

Additional file 3: **Movie S2.** The video clip showing the traveling of red blood cells in zTM-b morphant. (MOV 17000 kb)

Additional file 4: **Movie S3.** The video clip showing larval spontaneous movement. (MOV 72000 kb)

## Data Availability

All data generated or analyzed during the current study are included in this published article.

## Abbreviations

AA1: Mandibular aortic arch
APC: Activated protein C
CCV: Common cardinal veins
DA: Dorsal aorta
DEL: Migratory deep cells
dpf: Day post fertilization
EGF: Epidermal growth factor
EVL: Enveloping layer
Hep: Heparin
hpf: Hours post-fertilization
hTM: Human thrombomodulin
HV: Head vasculatures
ISV: Intersegmental blood vessels
MCeV: Mid-cerebral vein
MO: Morpholino oligonucleotides
MSV: Mesencephalic vein
OV: Optic vein
PHBC: Primordial hindbrain channel
PICA: Primitive internal carotid artery
pLL: Posterior lateral line
PMBC: Primordial midbrain channel
Thr: Thrombin
TM: Thrombomodulin
TM-a: Zebrafish TM-like protein-a
TM-b: Zebrafish TM-like protein-b
YCL: Yolk cytoplasmic layer
YSL: Yolk syncytial layer
YSN: Yolk syncytial nuclear
