# Supplementary material for: A thrombomodulin-like gene is crucial to the collective migration of epibolic blastomeres during germ layer formation and organogenesis in zebrafish
Source: J Biomed Sci. 2019 Aug 26;26:60. doi: 10.1186/s12929-019-0549-2 (PMC6709559; doi:10.1186/s12929-019-0549-2)
Supplement: Supplementary file 1 — Figure S1. The alignment and identity of zTHBD primary sequence with those of zebrafish TM-a and TM-b. Figure S2. Efficiency of zTM-b MO. Figure S3. Schematic diagram of the predictive ETS-1 binding sites in TM promoter. Figure S4. The zTM-b morphants did not recover from the developmental anomalies as the embryogenesis proceeding to the later stages. Figure S5. In vitro activity assay for thrombomodulin. (PDF 1830 kb) [file 12929_2019_549_MOESM1_ESM.pdf]

zTHBD vs. zTM-a

```
zTHBD  MRELVMALAFALLGVHANKYIGDCVDGKCFVVQTDTANFDAAQKVCEERTGHLMTIRTAK
zTM-a  MRELVMALAFALLGVHANKYIGDCVDGKCFVVQTDTANFDAAQKVCEERTGHLMTIRTAK
       ************************************************************

zTHBD  LADVLSRLLLKGESGNFWIGLRYVGNECSDRLKGYKWITGDDTSHFSNWKRESDCAPSCV
zTM-a  LADVLSRLLLKGESGNFWIGLRYVGNECSDRLKGYKWITGDDTSHFSNWKRESDCAPSCV
       ************************************************************

zTHBD  SVSQNDHKWTERPCSDRIDGYVCEYNNLNHCPPLSTDEKVHYSTAFGFTSNELLREIPEL
zTM-a  SVSQNDHKWTERPCSDRIDGYVCEYNNLNHCPPLSTDEKVHYSTAFGFTSNELLREIPEL
       ************************************************************

zTHBD  TNATRLDLRTKHICFDSEWLHAPWNCEVFDGGCDYTCVRNGQSYACICQPGFKLDTNGIS
zTM-a  TNATRLDLRTRHICFDSEWHHAPWNCEVFDGGCDYKCVKNGQSQSCICPPGFKLDTNGIS
       **********.******** ***************.**.**** :*** ***********

zTHBD  CSQQQDDDPCAHAGCEHECARDSGAFACTCRPGFTLSADGKSCKENCKPGYINENGGCVD
zTM-a  CSQQQDDDPCAHAGCEHECARDSGAFACTCRPGFTLSADGKSCKENCKPGFINENGGCVD
       **************************************************:*********

zTHBD  VDECATGPCEHDCANTEGSYQCVCFDGYKPAAKDMHKCKMHCPDMRCKAECDPNNDYQCD
zTM-a  VDECATGPCEHDCANTEGSYKCVCFDGYKPAAKDMHKCIMHCTDMRCKPECDPNNNYQCD
       ********************:***************** ***.*****.******:****

zTHBD  CPDGFLFDEDDRYCVDVDECISTNDCDQNCTNTPGSFECFCHEGYVLLDNGVCE--DLEG
zTM-a  CPDGFLFDEDDRYCVDVDECISTNTCDQNCTNTPGSFECFCYEGYVLLDNGVCEEQDLEG
       ************************ ****************:************  ****

zTHBD  SGSSTPFDLFIPTSRPPTDRPLSISAGSLLGVMVCVVVCVLLLVCLAHCILRRLSKTNHY
zTM-a  SGSSTPFDLFIPTSRPPTDRPLSISAGSLLGVMVCVVVCVLLLVCVAHCILRRLSKMNHY
       *********************************************:********** ***

zTHBD  DTHKVHDEIYDFQQVITENDSTQQSLPNTNRYLKRDI
zTM-a  DTHKVHDEIYDFQQVITENDSTQQSLPSTNRDLKRDI
       ***************************.*** *****
```

	zTHBD	zTM-a	zTM-b
zTHBD	100	96	20
zTM-a	-	100	25
zTM-b	-	-	100

zTHBD vs. zTM-b

```
zTHBD  --------MRELVMALAFALLGVHANKYI---------GDCVDGKCFVVQTDTANFDAAQ
zTM-b  MGKRADRKSCAVYPCLFFGLLYLYWSKGVMGQSLQEQDGMCDADGCYSIHLQRKTFRESS
                  :  .* *.** :: .* :         * *  . *: :: :  .*  :.

zTHBD  KVCEERTGHLMTIRTAKLADVLSRLLLKGESG------NFWIGLRYVGNECSDR--LKGY
zTM-b  RICKDKGGNLATIKGPEEASLIHNLLSSGVRRVPRPRIRLWIGLQRMPRQCSATRPLRGF
       .:*::. *:* **. .: *.:: .** .*         .:****. : .:**    *.*:

zTHBD  KWITGDDTSHFSNWKRE---SDCAPS-CVSVS----------QNDHKWTERPCSDRIDGY
zTM-b  TWSTGEQETQYTNWQRDDVPNACAAHRCVVISYNTADKSGSDQNNFKWLDGSCAVAVDGF
       .* **:: ::::**:*:   . **.  ** :*          **:.** : .*:  :**:

zTHBD  VCEYNNLNHCPPLSTD--EKVHYSTAFGFTSNELLREIPELTNATRLDLRTKH------I
zTM-b  LCSYTYQGMCPALQNEGGGPALYNTPFELAST-ILTHVP-FGSVANLPCREGEKEDQSVL
       :*.*.  . **.*..:    . *.*.* ::*. :*  :* : ..:.*  *         :

zTHBD  CFDSEWLHAPWN-------------CEVFDGGCDYTCVRNGQSYACICQPGFKLDTNGIS
zTM-b  CMVRDDGSIGWSKDAPLCTDIPKDLCEQDNGGCEHYCENRNTHYYCECSEGYVLGEDGKT
       *:  :     *.             **  :***:: * ...  * * *. *: *. :* :

zTHBD  CSQQQDDDPCAHAGCEHECARDSGAFACTCRPGFTLSADGKSCKE---------------
zTM-b  C---QLSDPCHRANCEFECESTAQGHHCKCPDGYLLSGDGQSCLDIDECLQKPCPQECIN
       *   * .*** .*.**.**   : .. *.*  *: **.**:** :

zTHBD  -------NCKPGYI-NENGGCVDVDECATGPCEHDCANTEGSYQCVCFDGYKPAAKDMHK
zTM-b  APGTFECRCNEGYLTSEFGECVDVDECMEGKCDHICENLNGSYTCLCHEGFSPLREDPDL
              .*: **: .* * *******  * *:* * * :*** *:*.:*:.*  :*

zTHBD  C----KMHCPDMRCKAEC-DPNNDYQCDCPDGFLFDEDDRYC--VDVDECISTNDCDQNC
zTM-b  CEDIDECKTPEI-CDQVCRNYEGGFECLCDEGYILQEDNYYCRPVEEDEHLTIQSADATM
       *    : : *:: *.  * : :..::* * :*::::**: **  *: ** :: :..* .

zTHBD  TNT------------------------PGSFECFCHEGYV------------------LL
zTM-b  NDPDLVTYASTTEWLTDAPSIEWHPTYPSWFTEKTEDGDVTTQEADLTEHAFSNSPDHLL
       .:.                        *. *     :* *                  **

zTHBD  D----------------NGVCEDLEGSGSST-----------------------PFDLFI
zTM-b  DITESNKEDEGISIATSDSVAARASGNKRTSEALTSAPKKNRVFVMSTSATSQLPEDALA
       *                :.*.   .*.  ::                       * * :

zTHBD  P-TSRPPTDRPLSISAGSLLGVMV--CVVVCVLLLVCLAHCILRRLSKTNHYDTHKVHDE
zTM-b  PGTQQRPGDKKKHDKSWLLVALLVPLCVFIVVMLALGIVYC------TSCAVEPRKSVTD
       * *.. * *.    .:  *:.::*  **.: *:* : :.:*      .:   :..*   :

zTHBD  IYDFQQVITENDSTQQSLPNTNRYLKRDI
zTM-b  CYRW---------TTTSKPEKSSTAKSQA
        * :         *  * *:..   * :
```

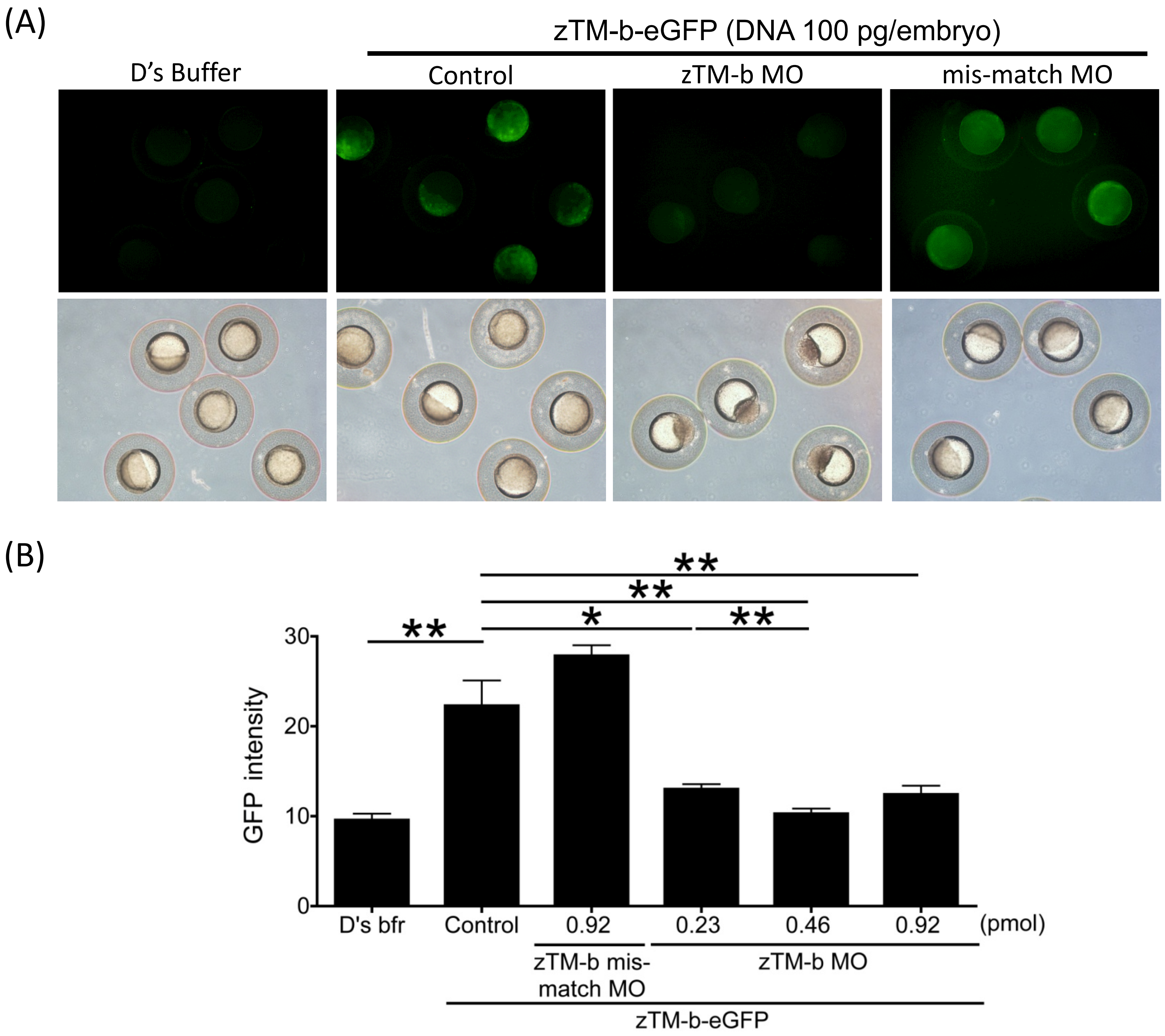
(A)
zTM-b-eGFP (DNA 100 pg/embryo)
D's Buffer
Control
zTM-b MO
mis-match MO
(B)
**
**
*
**
**
30
20
10
0
GFP intensity
D's bfr
Control
0.92
0.23
0.46
0.92
(pmol)
zTM-b mis-match MO
zTM-b MO
zTM-b-eGFP

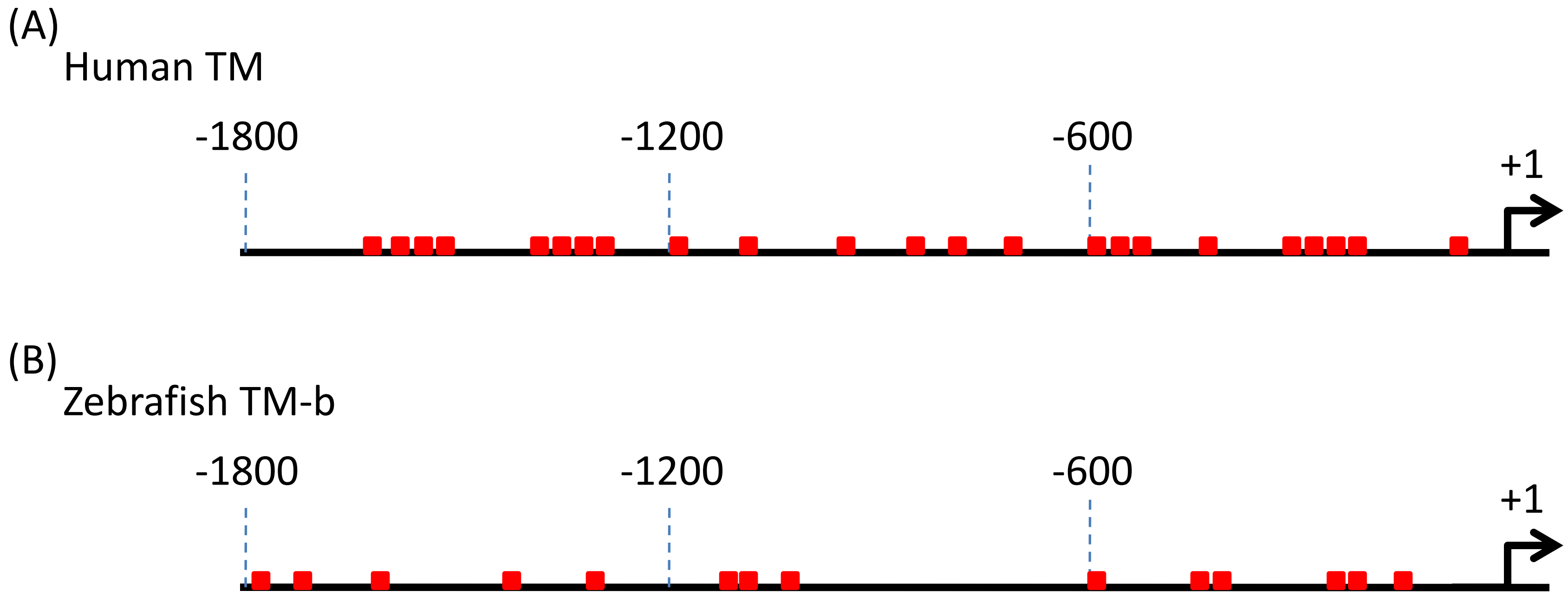

(A)
Human TM
-1800
-1200
-600
+1
(B)
Zebrafish TM-b
-1800
-1200
-600
+1

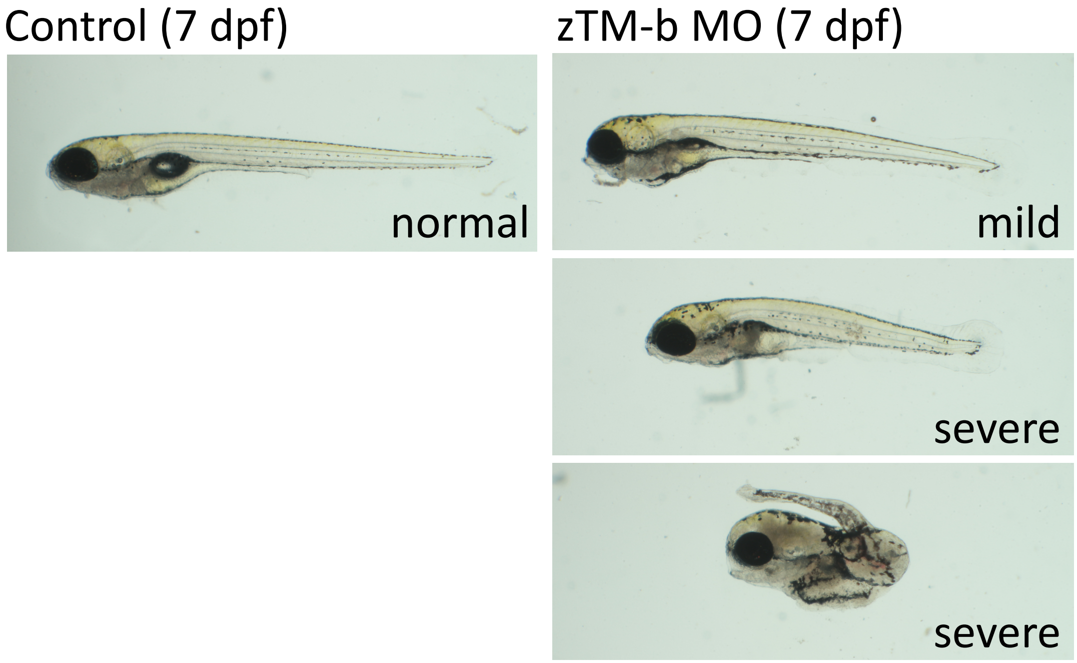
Control (7 dpf)
zTM-b MO (7 dpf)
normal
mild
severe
severe

(A)

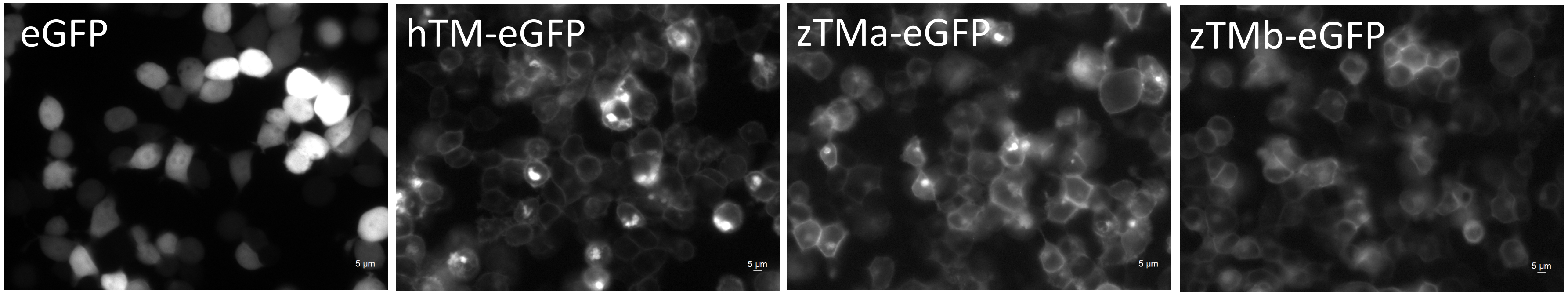
eGFP
hTM-eGFP
zTMa-eGFP
zTMb-eGFP

(B)

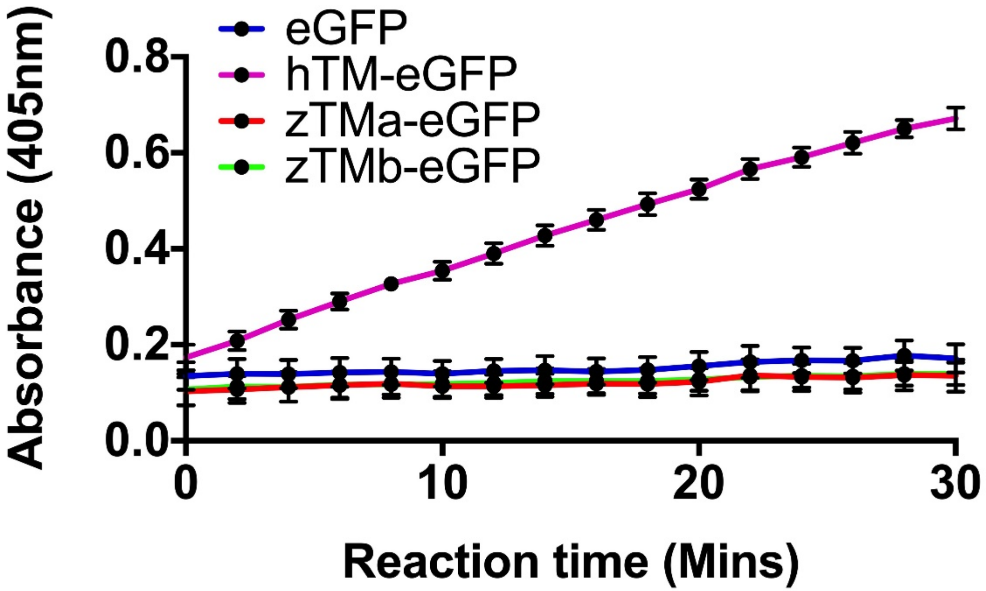
eGFP
hTM-eGFP
zTMa-eGFP
zTMb-eGFP
Absorbance (405nm)
0.0
0.2
0.4
0.6
0.8
0
10
20
30
Reaction time (Mins)
